# Comparison of VividTrac, King Vision and Macintosh laryngoscopes in normal and difficult airways during simulated cardiopulmonary resuscitation among novices

**DOI:** 10.1371/journal.pone.0260140

**Published:** 2021-11-18

**Authors:** Dóra Keresztes, Ákos Mérei, Martin Rozanovic, Edina Nagy, Zoltán Kovács-Ábrahám, János Oláh, Péter Maróti, Szilárd Rendeki, Bálint Nagy, Gábor Woth

**Affiliations:** 1 Department of Anesthesiology and Intensive Therapy, Medical School, University of Pécs, Pécs, Hungary; 2 Medical Skills Lab, Medical School, University of Pécs, Pécs, Hungary; 3 Department of Operational Medicine, Medical School, University of Pécs, Pécs, Hungary; 4 Medical School, University of Pécs, Pécs, Hungary; Stony Brook University Renaissance School of Medicine, UNITED STATES

## Abstract

**Introduction:**

Early endotracheal intubation improves neurological outcomes in cardiopulmonary resuscitation, although cardiopulmonary resuscitation is initially carried out by personnel with limited experience in a significant proportion of cases. Videolaryngoscopes might decrease the number of attempts and time needed, especially among novices. We sought to compare videolaryngoscopes with direct laryngoscopes in simulated cardiopulmonary resuscitation scenarios.

**Materials and methods:**

Forty-four medical students were recruited to serve as novice users. Following brief, standardized training, students executed endotracheal intubation with the King Vision^®^, Macintosh and VividTrac^®^ laryngoscopes, on a cardiopulmonary resuscitation trainer in normal and difficult airway scenarios. We evaluated the time to and proportion of successful intubation, the best view of the glottis, esophageal intubation, dental trauma and user satisfaction.

**Results:**

In the normal airway scenario, significantly shorter intubation times were achieved using the King Vision^®^ than the Macintosh laryngoscope. In the difficult airway scenario, we found that the VividTrac^®^ was superior to the King Vision^®^ and Macintosh laryngoscopes in the laryngoscopy time. In both scenarios, we noted no difference in the first-attempt success rate, but the best view of the glottis and dental trauma, esophageal intubation and bougie use were more frequent with the Macintosh laryngoscope than with the videolaryngoscopes. The shortest tube insertion times were achieved using the King Vision^®^ in both scenarios.

**Conclusion:**

All providers achieved successful intubation within three attempts, but we found no device superior in any of our scenarios regarding the first-attempt success rate. The King Vision^®^ was superior to the Macintosh laryngoscope in the intubation time in the normal airway scenario and noninferior in the difficult airway scenario for novice users. We noted significantly less esophageal intubation using the videolaryngoscopes than using the Macintosh laryngoscope in both scenarios. Based on our results, the KingVision^®^ might be recommended over the VividTrac^®^ and Macintosh laryngoscopes for further evaluation.

## Introduction

Airway management is a key intervention in every resuscitation attempt [1]. Tracheal intubation enables continuous, uninterrupted chest compressions with ventilation, and prevents gastric insufflation and gastric content aspiration into the lungs [2]. Additionally, Schuppen et al. reported that tracheal intubation reduces air leakage and improves patient safety during transport by reducing the probability of airway dislodgement [1]. However, in most cases, continuous chest compressions must be halted for the duration of the intubation attempt, which results in deterioration of the coronary blood flow and therefore decreases the effectiveness of cardiopulmonary resuscitation (CPR) [3]. Currently, the accepted gold standard device for endotracheal intubation (aside from coronavirus 2019 disease (COVID-19) infection-related case management) is the Macintosh-type bladed direct laryngoscope (DL) [4]. A critical point of the DL is the proper alignment of the vocal cords, oropharynx and oral cavity-mouth, which should be in a straight continuous line, allowing the insertion of the cuffed endotracheal tube [5]. A common maneuver to achieve the mentioned position is head-tilt manipulation, which cannot be used in situations of a probable or suspected cervical spine injury. To circumvent this and other airway management-related problems, various models of videolaryngoscopes (VLs) have been developed in recent years. These devices do not necessitate the alignment of airway-related structures and utilize a fiber-optic or optical lens system to provide airway visibility. Due to recent developments, one might observe an increase in the use of VLs with tube-guiding equipment [4–6]. According to recent literature, the use of VLs instead of DLs might improve the rate of intubation success on the first attempt among users with scarce experience using DLs or VLs [7–11]. In the hands of more experienced users, benefits have been observed, including a shorter duration of chest compression interruption [12].

The early successes of VLs has led to a broad diversity of available devices with different shapes, forms and sizes, but the effect of these differences on intubation success is not yet completely clear [6,10,13–17].

Considering the abovementioned data, our research group aimed to assess the effectiveness of DLs and various VLs in the hands of nonprofessional airway providers during simulated resuscitation situations. In emergency situations, airway management isoften performed by a less experienced physician, particularly undersuboptimal conditions [8]. Considering, that the first-attempt intubation success rate varies widely across VLs, our goal was to identify a device that is capable of supporting a successful first intubation attempt for novice users. In the era of COVID-19, the use of VLs has become a priority. In resuscitation, early intubation with a cuffed endotracheal tube is recommended to decrease the chance of COVID-19 transmission [18]. According to the most recent European Resuscitation Council (ERC) guidelines, if an advanced airway is required during resuscitation, only rescuers with a high endotracheal intubation success rate (95% within two attempts) should attempt endotracheal intubation [19]. For the sake of proper transmission prevention, single-use VLs with standalone monitors are recommended. These devices could sufficiently increase the distance between the provider and the airway of the patient. Although the VividTrac^®^ has not yet been evaluated in resuscitation scenarios, it completely fulfils the abovementioned criteria and has shown promising results [18,20]. Therefore, our aim was to evaluate the performance of novices with the VividTrac^®^ in standardized and safe resuscitation scenarios compared with the DL and King Vision^®^.

Our primary outcome was the rate of successful intubation, determined according to the most recent ERC guidelines of a >95% success rate within two attempts [19].

## Materials and methods

The study was carried out with the prior permission of the Institutional Scientific and Human Research Ethics Committee of the University of Pécs (7176 –PTE 2018). The investigation was performed at the Medical Skills Lab of the University of Pécs Medical School, Hungary. Based on our previous study, we defined both the devices to be examined and the required minimum sample size [20]. All participants provided written consent prior to the study. Participation in the study was voluntary and the participants were free to withdraw consent any time. Based on the results of our previous similar study, the following laryngoscopes were selected for evaluation [20]: the Macintosh bladed DL, size 4, (KaWe^®^, Asperg, Germany); (b) the VividTrac^®^ with an adult channeled hyperangulated blade (Vivid Medical, Palo Alto, USA); and the King Vision^®^ with a size 3 channeled standard blade (Ambu, Copenhagen, Denmark). To allow an ideal picture size for the provider, the VividTrac^®^ was connected to a 13.3” thin-film transistor screen panel. Since resuscitation itself increases the difficulty of airway management and we mainly aimed to compare the VividTrac^®^ to the gold standard DL, we performed calculations based on the „scenario B” intubation times of the VividTrac^®^(mean: 13.66 seconds) and the DL (mean: 20.93 seconds) [20]. With an enrollment ratio of 1:1, α = 0.05 and β = 0.1 we estimated a minimum sample size of 29. In total, we included 44 participants. As nonprofessionals without broad intubation experience, medical students were invited to participate in our study. Novice users took part in a 15-minute training sessions supervised by a consulting anesthesiologist and experienced investigators. During practice, participants acquired manual skills and theoretical knowledge regarding the use of each laryngoscope in the normal airway scenario (scenario A) and in the difficult airway scenario (scenario B). The utilization of optimization maneuvers, the use of a bougie, and the estimation of the percentage of glottic opening (POGO) score were also explained and practiced. Their attention was drawn to the mechanism and the relevance of dental injuries. Airway training was carried out on the Laerdal^**®**^ Airway Management Trainer (Laerdal^**®**^, Stavanger, Norway). The study was performed using an Advanced Life Support (ALS) simulator (Ambu^®^ Man Advanced) during continuous chest compressions performed by one of the study supervisors. Both the frequency and depth of chest compressions were in accordance with the protocol. This was verified by the provided Ambu^**®**^ ALS monitoring program. The simulator mannequin was placed in a hospital ward bed to simulate the challenge of a potentially moving a patient’s body during CPR. Two airway management scenarios were assessed: “in scenario A”, during continuous chest compressions, head tilting was allowed in “scenario B”, the cervical spine was immobilized with manual in-line stabilization (MILS) according to the Advanced Trauma Life Support algorithm. Each endotracheal intubation attempt was performed with a standard, cuffed, plastic, endotracheal tube 7.0 mm in internal diameter (Mallinckrodt^®^, Covidien, Dublin, Ireland). Participants were asked to perform intubation with each device in both scenarios in a random order. Randomization was performed by the closed envelope method, and participants selected the order immediately before the scenarios. The primary endpoint was successful endotracheal intubation. Additional endpoints included the number of intubation attempts, laryngoscopy time, tube insertion time, and intubation time; furthermore, the best achieved POGO was determined. We recorded esophageal intubation, dental injury, and bougie use. The laryngoscopy time was defined from when the laryngoscope blade passed through the interdental line until the achievement of the best POGO indicated by the beginning of tube manipulation. The intubation time ranged from the passage of the tube through the interdental line to successful intubation. The tube insertion time was defined as the difference between the exploration and intubation times. Attempt failure was defined as follows: the attempt took more than 120 seconds; the tube was removed from the oral cavity; or esophageal intubation occurred (recognized by the participant). Intubation failure was defined as 3 unsuccessful intubation attempts, failure of the participant to recognize esophageal intubation, or attempt resignation by the participant. The participant could not ask to for the chest compressions to be stopped during the intubation attempts.

After each scenario, we asked the participants to rate the device in the relevant scenario without ranking based on the ease of technical use (1 = easy, 5 = difficult), ease of physical use (1 = easy, 5 = difficult) and willingness to reuse (1 = would never use again, 5 = would like to use).

### Statistical analysis

Analyses were conducted using Statistical Package for the Social Sciences (SPSS) Statistics software, version 22.0 (IBM Corporation, Armonk, NY, USA). Continuous and ordinal data are presented as the median and interquartile range (IQR), while categorical data are presented as raw numbers and frequencies. Nonparametric tests were used since the data were not normally distributed, including the Kolmogorov-Smirnov and Shapiro-Wilk tests. The Kruskal-Wallis test by ranks (one-way ANOVA on ranks) with Dunn’s post hoc test was used to assess pairwise differences between devices regarding the following variables: laryngoscopy time, tube insertion time, intubation time, POGO score, ease of technical use, ease of physical use and willingness to reuse. Chi-square tests were used to evaluate differences in categorical data among devices regarding the rate of successful tracheal intubation, esophageal intubation, dental injury, and bougie usage. Values of P < 0.05 were considered significant.

## Results

Data regarding “scenario A” are shown in detail in Table 1.

**Table 1:** Results of “Scenario A”.

Scenario A	DL (n = 44)	KV (n = 44)	VT (n = 44)
**Number of attempts (n, 1/2/3)**	30/8/6	35/6/3	37/5/2
**Laryngoscopy time (s)**	10.09 [7.57–13.35]	9.36 [7.31–14.91]	9.3 [6.05–15.13]
**Tube insertion time (s)**	7.4[5.84–14]	3.35 [2.33–8.7]^**¶**^	11.69 [5.3–19.61]^**†**^
**Intubation time (s)**	19.19 [14.28–27.09]^**†**^	15.2 [11.1–23.9]*^¶^	23.08 [15.9–33.2]^**†**^
**POGO (%)**	75 [60, 90]	75 [70–80]	60 [50–90]
**Ease of technical use (1–5)**	2 [1–4]	3 [2–4]	2 [2–4]
**Ease of physical use (1–5)**	3 [2–4]	2 [2–3]	2 [1–3]
**Willingness of reuse (1–5)**	4 [3–5]	3 [2–4]	4 [2–5]
**Use of bougie (n)**	11^**¶**^^**†**^	0*	0*
**Dental injury (n)**	0	1	0
**Esophageal intubation (n)**	11^**¶**^^**†**^	1*	1*

Data are reported as the median [IQR] or as numbers (n).

*Significant difference (P < 0.05) compared to the DL.

†Significant difference (P < 0.05) compared to the KV.

¶Significant difference (P < 0.05) compared to the VT. DL: Direct laryngoscope (Macintosh), KV: King Vision^®^, POGO: Percent of glottic opening, VT: VividTrac^®^.

We did not register unsuccessful intubation in “scenario B”. The rate of success within two attempts was 86.4%, 93.2% and 95.5% using the DL, King Vision^®^ and VividTrac^®^, respectively. There were no significant differences in the number of intubation attempts or the best POGO score achieved. The King Vision^®^ was faster regarding the tube insertion time than the VividTrac^®^ (P < 0.05). The King Vision^®^ was superior to the VividTrac^®^ and DL in terms of the intubation time. There were no significant differences in the laryngoscopy time and tube insertion time between the VLs and DL. The participants recognized all cases of esophageal intubation. The incidence of esophageal intubation and bougie use was higher with the DL (P < 0.05). Overall, only one dental injury occurred in this scenario. The score of neither the ease of use nor the willingness to reuse was significantly different among the devices.

Data regarding “scenario B” are shown in detail in Table 2.

**Table 2:** Results of “Scenario B”.

Scenario B	DL (n = 44)	KV (n = 44)	VT (n = 44)
**Number of attempts (n, 1/2/3)**	35/4/5	40/3/1	35/2/7
**Laryngoscopy time (s)**	13.7 [8.37–18.89]^**¶**^	14.52 [10.72–26.05]^**¶**^	8.04 [6.33–14.33]*^†^
**Tube insertion time (s)**	8.15 [4.4–17.09]^**†**^	4.76 [2.05–11.42]*^¶^	8.09 [4.03–18.64]^**†**^
**Intubation time (s)**	23.39 [16.93–34.31]	21.91 [14.76–39.51]	20.83 [12.65–39.45]
**POGO (%)**	60 [40–80]	67.5 [50–80]	60 [40–76.3]
**Ease of technical use (1–5)**	3 [2–4]	3[2–4]	2 [1–4]
**Ease of physical use (1–5)**	4 [2–4]^**¶**^	3 [2–4]	2 [1–3]*
**Willingness of reuse (1–5)**	3 [2–5]	3 [2–4]	4 [2–5]
**Use of bougie (n)**	17^**¶**^^**†**^	0*	0*
**Dental injury (n)**	0	0	0
**Esophageal intubation (n)**	7^**¶**^^**†**^	0*	0*

Data are reported as the median [IQR] or as numbers (n).

*Significant difference (P < 0.05) compared to the DL.

†Significant difference (P < 0.05) compared to the KV.

¶Significant difference (P < 0.05) compared to the VT. DL: Direct laryngoscope (Macintosh), KV: King Vision^®^, POGO: Percent of glottic opening, VT: VividTrac^®^.

We did not register unsuccessful intubation in „scenario B”. The rate of success within two attempts was 88.6%, 97.7% and 84.1% using the DL, King Vision ^®^ and VividTrac^®^, respectively. There were no significant differences in the number of intubation attempts or the best POGO score achieved. The laryngoscopy time was shorter with the VividTrac^®^ than the King Vision^®^ and DL (P < 0.05), but the King Vision^®^ was superior to the VividTrac^®^ and DL in terms of the tube insertion time. There were no significant differences in the intubation time among the devices. The VividTrac^®^ received a better ease of physical use score than the DL (P < 0.05) and King Vision^®^, but the scores for the ease of technical use and willingness to reuse were similar. Dental injury did not occur. The incidence of esophageal intubation and bougie use was higher with the DL (P < 0.05). The participants recognized all cases of esophageal intubation.

## Discussion

Before detailed discussion of our results, the following limitations should be considered. All data were obtained from a monocentric mannequin study in which interventions were performed by medical students. The mannequin model may not precisely reproduce the intubation and resuscitation conditions of real patients. The time gap between the training and evaluation phases of the study was thirty minutes; therefore, the transferability of our findings into clinical practice is questionable. The training and measurement took place on two different simulators. Participants’ intubation skills on the ALS simulator probably improved from scenario A to scenario B. Furthermore, dental trauma was assessed in a “yes” or “no” fashion, regardless of the exact number of “clicks” experienced during the attempts. MILS could decrease the effect of CPR on head and laryngeal structure movement and thus might affect differences between scenario A and B regarding airway difficulty.

The success of CPR is highly dependent on the effectiveness of chest compressions and their necessary interruptions. A longer chest compression interruption would deteriorate the overall CPR outcome [3]. In the hands of unexperienced providers, a DL would result in longer compression interruptions and multiple intubation attempts [9]. The use of a VL improved the Cormack-Lehane classification of the laryngoscopic view and decreased the number of intubation attempts in these scenarios [13,21]. Our study assessed the effectiveness of the King Vision^®^ and VividTrac^®^ during intubation by unexperienced users in a simulated reanimation situation. Previous studies have concluded that the type of laryngoscope used for intubation during CPR is not a significant factor of success among experienced users [11]. The recent literature is inconclusive regarding this question, as some former data have suggested that certain VLs are more suitable in this scenario, while other publications have reported no differences in benefits among different equipment [8,10,22].

The selected VLs have not been previously assessed. According to data in the literature, with using a DL, approximately 5.8% of cases are difficult airway cases, while during emergency situations (including CPR), 14.8% of cases are difficult [10]. In-line stabilization of the cervical spine can be used to simulate a difficult airway situation. All values for the LEMON criteria (mouth opening, modified Mallampati classification, and neck extension) worsen significantly after cervical collar application [23]. Literature regarding intubation by unexperienced providers in difficult airway situations during CPR are scarce, and the two VLs assessed herein have not been assessed in these scenarios before. According to our criteria, we did not register a failed intubation attempt with any device in any scenario. However, an acceptable success rate according to the most recent ERC guidelines (>95% within two attempts) was only achieved using the VividTrac^®^ in “scenario A” and the King Vision^®^ in “scenario B”. In concordance with Han et al., we found a significantly higher esophageal intubation rate with the DL than with the VLs [8]. We found no difference in the number of intubation attempts between the DL and each VL in the simulated CPR intubation environment. A study by Gaszynska et al. was unable to demonstrate a significant difference between the King Vision^®^ and DL [22], while other studies have found better success rates for particular VLs [7–10]. In the case of a difficult airway, the difference is also notable among experienced users [17].

A significant difference was not found among the laryngoscopes in the normal airway situation, while the VividTrac^®^ yielded a significantly shorter laryngoscopy time than the other tools in the difficult airway situation. The King Vision^®^ yielded a significantly shorter tube insertion time. Additionally, in “scenario A”, the King Vision^®^ yielded a shorter intubation time than the VividTrac^®^ and DL. In “scenario B”, no VL proved to be superior. Szarpak et al. and Han et al. formerly reported shorter laryngoscopy and intubation times with VLs than DLs [8,17], while there is controversy regarding the King Vision^®^ compared to a DL [22].

The benefit of VLs in the hands of experienced users is still questionable. Earlier studies provided evidence that the use of certain VLs comes with the benefit of significantly shorter intubation time, while others did not show improvement even in the hands of unexperienced providers compared to DLs. In the case of experienced providers, it is more difficult to provide evidence for the use of VLs. The main difficulty of DLs is proper alignment of the mouth-oropharynx-glottic opening [5,24]. This procedure is not required during VL use; thus, former studies have reported better visualization results with VLs during CPR, independent of user experience [8,11,17]. In contrast, our results during constant chest compressions in both scenarios do not support this conclusion. While dental injury was not registered during the intubation attempts, the incidence of esophageal intubation was greater with the DL, which is in accordance with former results [9–11]. This might result from the possibility of visual checks during tube insertion with a VL [8]. The use of elastic tube guidance was significantly higher with the DL, although this might result from the fact that all VLs had tube-guiding sheaths.

Our previous study demonstrated easier technical and physical use of VLs than DLs [20]. This result is in concordance with the findings reported by Han et al., who also conducted a CPR study under uninterrupted chest compressions [8]. In contrast to these findings, we noted a significant difference only between the DL and VividTrac^®^ in “scenario B”. In studies where the number of intubation attempts was lower and users found the use of a VL subjectively superior to that of a DL, an increased POGO score and lower incidence of intubation-related complications were registered [8]. Currently, an increasing number of VLs have become available on the market however, their real benefit during emergency intubation situations is not completely clear. The significant heterogeneity of the studied patient populations and study approaches could be a cause of the differences in the results of VL effectiveness [25]. Additionally, the experience of the studied providers might play a key role in these discrepancies.

## Conclusion

All providers achieved successful intubation within three attempts in our study. While we found no device superior in any of our scenarios regarding the first-attempt success rate, the ERC criteria were met in the normal airway scenario only by the VividTrac^®^ and in the difficult airway scenario only by the King Vision^®^. The King Vision^®^ was superior to the Macintosh laryngoscope in terms of the intubation time in the normal airway scenario and noninferior in the difficult airway scenario among novice users. We noted significantly fewer instances of esophageal intubation by the VLs than the Macintosh laryngoscope in both scenarios. Based on our results, the KingVision^®^ might be recommended over the VividTrac^®^ and Macintosh laryngoscopes for further evaluation.

## Supporting information

S1 Dataset (XLSX)
